# Moral foundations, values, and judgments in extraordinary altruists

**DOI:** 10.1038/s41598-022-26418-1

**Published:** 2022-12-21

**Authors:** Paige Amormino, Montana L. Ploe, Abigail A. Marsh

**Affiliations:** Department of Psychology, Georgetown University, 304 White-Gravenor Hall, 3700 O Street N.W., Washington, DC, 20057 USA

**Keywords:** Psychology, Human behaviour

## Abstract

Donating a kidney to a stranger is a rare act of extraordinary altruism that appears to reflect a moral commitment to helping others. Yet little is known about patterns of moral cognition associated with extraordinary altruism. In this preregistered study, we compared the moral foundations, values, and patterns of utilitarian moral judgments in altruistic kidney donors (*n* = 61) and demographically matched controls (*n* = 58). Altruists expressed more concern only about the moral foundation of harm, but no other moral foundations. Consistent with this, altruists endorsed utilitarian concerns related to impartial beneficence, but not instrumental harm. Contrary to our predictions, we did not find group differences between altruists and controls in basic values. Extraordinary altruism generally reflected opposite patterns of moral cognition as those seen in individuals with psychopathy, a personality construct characterized by callousness and insensitivity to harm and suffering. Results link real-world, costly, impartial altruism primarily to moral cognitions related to alleviating harm and suffering in others rather than to basic values, fairness concerns, or strict utilitarian decision-making.

## Introduction

In 2021, 4,905 patients on the United States transplant list died while waiting for a kidney transplant^1^. That’s the bad news. The good news is that 473 people were saved from a similar fate by donations from unrelated, anonymous, living kidney donors, who underwent surgery to give a stranger their own kidney^1^. Such donations meet the most stringent definitions of altruism, as they entail non-normative, non-reciprocal, anonymous, and costly helping to benefit a stranger^2^. Consistent with this, extraordinary altruists preferentially engage in various other forms of altruism, including blood donation, volunteering, and sharing money with strangers^3,4^. Altruistic kidney donation also appears to reflect unusual moral values or patterns of judgments, such as heightened concern about reducing or avoiding harm, or unusually utilitarian moral reasoning that prioritizes reducing suffering in an impartial manner. But no prior study has quantified moral foundations, values, and patterns of moral judgments that correspond to real-world extraordinary altruism. In this preregistered study, we aimed to investigate these features in a rare sample of non-directed living kidney donors to identify patterns of moral cognition that may support this form of extraordinary altruism, and—in that altruistic kidney donations satisfy stringent definitions of altruism—perhaps altruistic behavior more generally.

Morality refers to convictions regarding what is right or wrong^5,6^. These convictions are driven by fundamental values that guide moral judgment^5,7^. Moral judgments can take many forms, however, which reflect a variety of concerns and convictions. According to *moral foundations theory*, five universal foundations shape judgments of morality: harm/care, fairness/reciprocity, ingroup/loyalty, authority/respect, and purity/sanctity^8–11^. These five foundations include individualizing values (harm/care and fairness/reciprocity) which reflect concern for the rights and well-being of individuals, and binding values (ingroup/loyalty, authority/respect, and purity/sanctity) which reflect concern for factors that promote the welfare of groups^12–14^.

These various values can in some cases promote conflicting outcomes^15–19^. For example, choosing between spending time with a relative versus volunteering to help strangers^20^ could bring the values of harm/care and fairness/reciprocity into conflict with ingroup/loyalty^16^. Similarly, non-directed kidney donation satisfies values related to impartial harm/care and fairness/reciprocity by reducing suffering without consideration of the beneficiary’s relationship to the donor. But such donations may violate moral foundations related to ingroup/loyalty (by, for example, potentially imposing costs on family members to benefit a stranger during the donation process and leaving donors unable to donate to relatives in the future) and purity/sanctity (by, for example, violating the wholeness of the body)^21–23^. To date, however, no one has assessed how moral foundations relate to the choice to perform this act of costly, impartial altruism.

Prior work suggests that in the laboratory, impartial prosociality is associated with the individualizing values of harm/care and fairness/reciprocity. Respondents who more strongly endorse these values are more cooperative and trusting with strangers in economic exchange games^14^, attend to and report feeling more responsible for strangers’ suffering^24^, are more likely to volunteer for charity^25^ and donate more to causes benefiting out-groups^26^. By contrast, respondents who more strongly endorse binding values are less likely to volunteer for or donate to charitable causes^25^, attend less to suffering strangers, and feel less moral responsibility for them^24^. But research linking moral foundations to non-self-reported, real-world prosocial behavior is scant^25^. In light of the available laboratory-based evidence, we predicted that extraordinary real-world altruists would endorse individualizing foundations—harm/care and fairness/reciprocity—more than controls.

Morality is thought to be (at least partially) motivated by values, which are goals and principles that people use to navigate through life^27,28^*.* At least ten distinct basic motivational values^27^ are verified cross-culturally—including power, achievement, hedonism, stimulation, self-direction, universalism, benevolence, traditionalism, conformity, security—that may contribute to universal moral foundations. For example, the values of universalism (defined as “understanding, appreciation, tolerance, and protection for the welfare of all people and for nature”^29^) and benevolence (defined as “preserving and enhancing the welfare of those with whom one is in frequent personal contact”^29^) are linked to endorsing harm/care and fairness/reciprocity^30^. If costly altruism for strangers is associated with harm/care and fairness/reciprocity, it may be associated with both values of universalism and benevolence. Accordingly, endorsements of universalism have been linked to cooperation and trust in economic exchange games^14,31^. Note, however, that universalism and benevolence may only be related moderately and marginally, respectively, to actual everyday prosocial behaviors^32^. We predicted that extraordinary altruism for strangers would be most strongly associated with universalism, which emphasizes impartiality in care, as we have found altruists respond altruistically at similar rates for socially close and distant others^4^. We also predicted that extraordinary altruism for strangers would be associated with benevolence, which also emphasizes concerns regarding welfare for others. Finally, we predicted costly altruism for strangers would be associated with reduced endorsement of self-enhancing values like power (defined as “social status and prestige, control or dominance over people and resources”^29^) and hedonism (defined as “pleasure or sensuous gratification for oneself”^29^) based on past research finding empathy to be negatively correlated with such values^33^.

Such values and moral concerns help shape moral judgments, the process by which people arrive at decisions in the moral realm. Past work has primarily examined moral judgments from two perspectives: *utilitarianism*—using the overarching goal of maximizing welfare to guide decisions—and *deontology*—using values such as human rights and duties to guide decisions, regardless of utilitarian consequences^34–37^. The impartial generosity towards strangers that characterizes extraordinary altruism most closely aligns with the principle of maximizing welfare for others regardless of their identity or relationship to the self^38–40^. At least when compared to not donating a kidney, donation maximizes the combined welfare of both altruist and recipient by (usually) inflicting only minimal harm on the donor while saving the recipient’s life. This suggests altruists may be characterized by a preference for utilitarian reasoning. But the utilitarian goal of maximizing welfare for others regardless of their identity or relationship to oneself can, when taken to an extreme, result in other important moral values being violated.

For example, harming one person to help several others would maximize welfare but would violate deontological values of individual rights. Causing harm to achieve utilitarian goals is qualitatively different from helping in which nonconsensual harm does *not* occur—for example, sacrificing oneself to benefit others. The Oxford Utilitarianism Scale (OUS)^37^ distinguishes these aspects of utilitarianism via two subscales: impartial beneficence, which reflects maximizing welfare in an impartial, self-sacrificial manner, and instrumental harm, which reflects maximizing welfare in a manner that allows for harming others. Prior work finds that people who endorse impartial beneficence also endorse valuing close and distant others equally, whereas people who endorse instrumental harm preferentially endorse empathizing with members of some groups over others^41^. To our knowledge, little data exists linking utilitarianism to real-world prosocial behaviors outside the laboratory. We predicted that extraordinary altruists would endorse impartial beneficence more highly than controls but would not endorse instrumental harm.

### The current research

In sum, we predicted that, relative to controls, extraordinary altruists would more strongly value the moral foundations of harm/care and fairness/reciprocity but would place less value on ingroup/loyalty and purity/sanctity. We also predicted they would more strongly value universalism and benevolence, but value power and hedonism less. We predicted that altruists would not differ from controls in their endorsement of conformity, following prior evidence that extraordinary altruists engage in similar rates of socially normative altruistic behaviors as controls^2,4^. Finally, we predicted that compared to controls, altruists would more strongly endorse impartial beneficence but less strongly endorse instrumental harm.

These predictions were informed by the available literature linking these forms of moral cognition to low-cost self-reported altruism in the lab and daily life. They were also informed by the available literature on moral cognition in psychopathy. Psychopathy is a personality construct associated with interpersonal callousness and insensitivity to others’ suffering and distress^42,43^. It is also associated with reduced endorsement of harm/care and fairness/suffering^44^, reduced valuation of universalism and benevolence^28^, increased valuation of power and hedonism^28^, and reduced impartial beneficence^37^. Prior research indicates that extraordinary altruists show “anti-psychopathic” patterns of empathic responding^45–47^. We thus assessed psychopathy in our sample as well to allow us to directly assess whether extraordinary altruism reflects “anti-psychopathic” patterns of moral cognition.

To test our predictions, we assessed endorsement of moral foundations, values, and utilitarian moral judgments in a sample of altruistic kidney donors and controls, using methodology similar to Glenn and colleagues’^28^ prior assessment of values and motivations in psychopathy We preregistered our planned sample size, predictions, and analyses at https://osf.io/xk4va/?view_only=4faad13131eb400983f065e3e2d9a22e. All analyses are thus confirmatory unless otherwise specified.

## Results

Our sample contained 61 altruistic kidney donors and 58 controls who were recruited and screened to be statistically comparable in terms of age, race, gender, and education level (see Supplementary Table S1 online). Because of known relationships among these demographic variables and patterns of moral cognition, we nevertheless included age, gender, and education in our analyses. Multicollinearity tests confirmed that in all models, variance inflation factor values were acceptable (< 5). Data were collected from January 22, 2021 to April 28, 2021 and analyzed using *R*. De-identified data and analyses are available on https://osf.io/xk4va/?view_only=4faad13131eb400983f065e3e2d9a22e. Descriptive statistics of all predictor variables by group can be found online in Supplementary Table S2.

### Moral foundations

**Figure 1:**
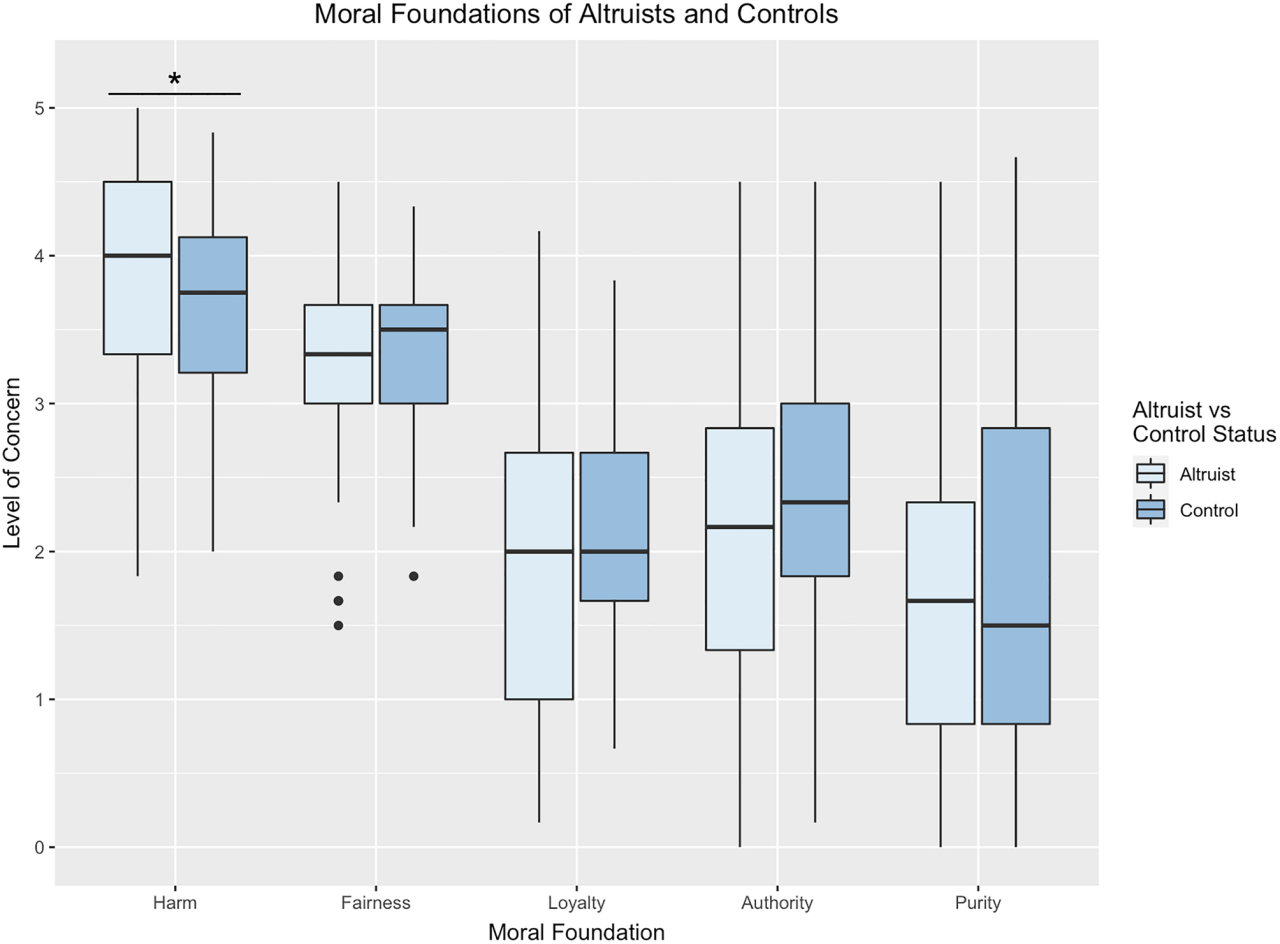
Boxplot of moral foundation scores for altruists and demographically matched controls. Note. *P < .05; **P < .01; ***P < .001.

We first assessed which moral foundations distinguish altruists from controls using a single regression model with group (altruist, control) as the outcome variable and the five moral foundations included as a predictor variables, while controlling for gender, age, and education. We used this approach to identify whether the unique variance associated with each foundation was associated with altruism while accounting for the other foundations. Results indicated harm/care was the only significant predictor of altruism (*OR* = 2.17, *95% CI* = [1.08, 4.34], *P* = 0.029), with every one-unit increase in harm/care increasing the likelihood of being an altruist by 117% (Fig. 1; Table 1). A dominance analysis confirmed that harm/care provided the highest average contribution (*r2.m* = 0.026)^48^. This pattern was broadly consistent with the results of bivariate correlation analyses (see Supplementary Table S3 online).

**Table 1:** Results from Multiple Logistic Regression Models Predicting Altruist Status from (1) Moral Foundations, (2) Schwartz Values, and (3) Oxford Utilitarianism, all Controlling for Sociodemographic Variables.

Factor	*b* (SE)	*OR* [*95%* *CI*]
**Moral foundations**		
Harm	0.77 (0.35)*	2.17 [1.08, 4.34]
Fairness	− 0.54 (0.38)	0.58 [0.278, 1.23]
Loyalty	− 0.10 (0.35)	0.90 [0.46, 1.79]
Authority	− 0.17 (0.34)	0.84 [0.43, 1.66]
Purity	− 0.06 (0.23)	0.94 [0.60, 1.49]
*Demographic covariates*		
Age	− 0.002 (0.02)	1.00 [0.96,1.04]
Education	0.01 (0.22)	1.01 [0.66, 1.54]
Gender^a^	0.09 (0.40)	1.09 [0.50, 2.40]
**Schwartz values**		
Power	− 0.15 (0.17)	0.86 [0.62, 1.21]
Hedonism	0.22 (0.22)	1.25 [0.1, 1.92]
Universalism	− 0.02 (0.20)	1.02 [0.69, 1.50]
Benevolence	− 0.002 (0.30)	1.00 [0.55, 1.80]
Conformity	− 0.05 (0.19)	0.95 [0.65, 1.38]
*Demographic covariates*		
Age	0.004 (0.02)	1.01 [0.97, 1.04]
Education	0.06 (0.21)	0.99 [0.70, 1.61]
Gender^a^	− 0.03 (0.39)	0.99 [0.45, 2.07]
**Oxford utilitarianism**		
Impartial Beneficence	0.50 (0.18)**	1.65 [1.16, 2.34]
Instrumental Harm	− 0.04 (0.18)	0.97 [0.68, 1.36]
*Demographic covariates*		
Age	− 0.0002 (0.02)	1.00 [0.96, 1.04]
Education	− 0.06 (0.21)	0.94 [0.63, 1.42]
Gender^a^	− 0.19 (0.39)	0.82 [0.38, 1.76]

Altruists were coded as ‘1’ and controls were coded as ‘0’.

^a^0 = female; 1 = male.

^+^*P* < .1; *P < .05; **P < .01; ***P < .001.

### Schwartz values

We next assessed basic values that distinguished altruists from controls. We again ran a simultaneous logistic regression with group (altruist, control) as the outcome variable and Schwartz values included as predictor variables, controlling age, gender, and education. Per recommendations by Schwartz^49^, our model only included values relevant to our preregistered hypotheses (power, hedonism, benevolence, universalism, conformity; Table 1), as including all 10 values in a single model could yield uninterpretable predictor weights^49^. This model identified no significant predictors. In an exploratory analysis, we also ran a logistic regression with group (altruist, control) as the outcome variable and the other five Schwartz values included as a predictor variable (achievement, stimulation, self-direction, security, traditionalism), controlling for age, gender, and education (see Supplementary Table S4 online). We found that altruists reported valuing traditionalism more strongly than controls (*OR* = 1.43, *95% CI* = [1.02, 2.01], *P* = 0.005) and controls valuing security more strongly than altruists (*OR* = 0.54, *95% CI* = [0.35, 0.83]*, P* = 0.036). Thus, for every one-unit increase in valuing traditionalism, the odds of being an altruist increased by 43%, and for every one-unit increase in valuing security, the odds of being an altruist decreased by 46%.

### Patterns of moral cognition

**Figure 2:**
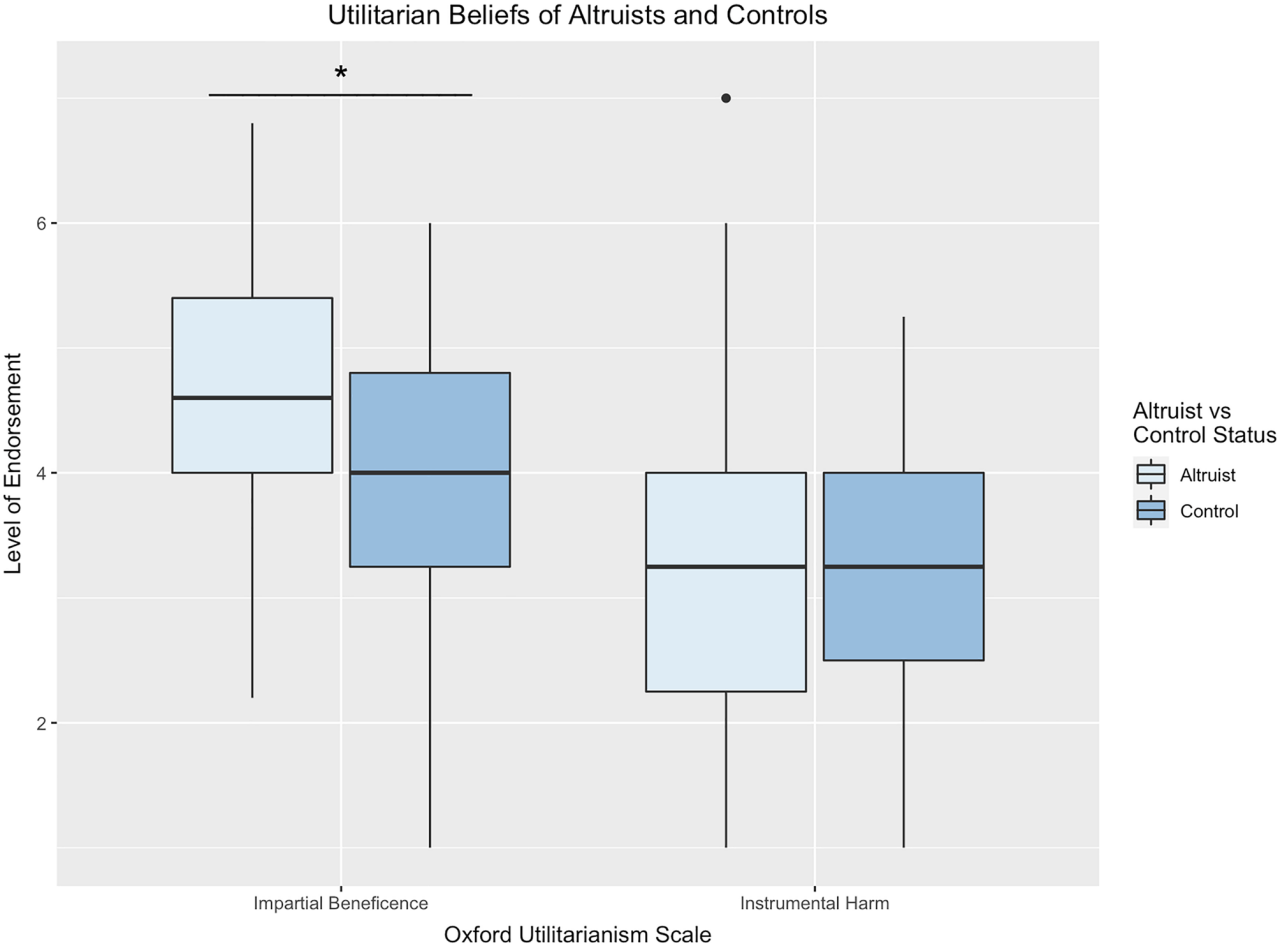
Boxplot of Oxford utilitarianism subscales for altruists and demographically matched controls. Note. ^+^*P* < .1; *P < .05; **P < .01; ***P < .001.

Next, we assessed patterns of moral judgments that distinguish altruists and controls using a simultaneous logistic regression with group (altruist, control) as the outcome variable and each utilitarian subscale (impartial beneficence and instrumental harm) included as predictor variables, controlling age, gender, and education. Results indicated that altruists more strongly endorse impartial beneficence (*OR* = 1.65, *95% CI* = [1.16, 2.34], *P* = 0.005; Fig. 2; Table 1). For every one unit increase in impartial beneficence, the odds of being an extraordinary altruist increased by 65%. Instrumental harm was not associated with altruism (*OR* = 0.97, *95% CI* = [0.68, 1.36], *P* = 0.840). Dominance analysis of this model confirmed that impartial beneficence had the highest average contribution (*r2.m* = *0.052*)^47^.

### Altruism and psychopathy

We next sought to assess whether altruism reflects “anti-psychopathic” patterns of moral cognition. We first confirmed that extraordinary altruism is associated with reduced psychopathy. We performed two separate logistic regressions to address this question: one predicting altruism from total LSRP scores and one predicting altruism from total TriPM scores (controlling for gender, age, and education; Table 2). We did not include multiple measurements of the same construct––the LSRP and the TriPM––in a single model because they are so strongly correlated (*r* > 0.50; see Supplementary Table S3 online for all bivariate correlations). Results indicated that lower LSRP scores predicted altruism, such that for every one-unit increase in psychopathy, the odds of being an altruist decreased by 84% (*OR* = 0.16, *95% CI* = [− 3.32, − 0.51], *P* = 0.0097). We found a trend-level association between altruism and TriPM total scores, such that for every one-unit increase in total psychopathy, the odds of being an altruist decreased by 75% (*OR* = 0.25, *95% CI* = [− 3.06, 0.15], *P* = 0.085) (Table 2). Results from separate logistic regressions with LSRP and TriPM subscales as predictors can be found on Table 2. Next, following Glenn and colleagues^28^, we conducted a series of linear regressions for each psychopathy subscale and total scores for both the LSRP and TriPM, controlling for age, gender, and education, and running separate models for each of our independent variables (see Supplementary Table S5 online). Results indicated that higher psychopathy was associated with lower endorsement of harm/care when using either the TriPM (*b* = − 0.87, *SE* = 0.25*, 95% CI* = [− 1.75, − 0.37], *P* = 0.008) or LSRP (*b* = − 0.89, *SE* = 0.20, *95% CI* = [− 1.29, − 0.50],* P* < 0.0001). Psychopathy was also associated with reduced endorsement of fairness/reciprocity using LSRP total scores (*b* = − 0.53, *SE* = 0.19, *95% CI* = [− 0.91, − 0.15], *P* = 0.007).

**Table 2:** Multiple logistic regressions predicting altruist status from psychopathy scores controlling for sociodemographic variables.

Factor	*b* (SE)	*OR* [*95%* *CI*]
**LSRP total**	− 1.84 (0.71)**	0.16 [− 3.32, − 0.51]
LSRP Primary	− 1.96 (0.68)**	0.14 [− 3.40, − 0.70]
LSRP Secondary	0.53 (0.44)	0.59 [− 1.42, 0.33]
**TriPM total**	− 1.39 (0.81)^+^	0.26 [− 3.06, 0.15]
TriPM Boldness	0.44 (0.41)	1.55 [− 0.37, 1.27]
TriPM Meanness	− 1.75 (0.60)**	0.17 [− 3.02, − 0.65]
TriPM Disinhibition	− 0.78 (0.53)	0.46 [− 1.88, 0.21]

Altruists were coded as ‘1’ and controls were coded as ‘0’. Beta values and standard errors are from multiple logistic regression models predicting altruist status from a single psychopathy sub- or total score while controlling for age, gender, and education.

^+^*P* < .1; *P < .05; **P < .01; ***P < .001.

We also assessed relationships between Schwartz Values and both psychopathy measures. LSRP total scores were associated with lower valuation of achievement (*b* = − 0.73, *SE* = 0.36, *95% CI* = [− 1.46, − 0.01], *P* = 0.047; see Supplementary Table S5 online), universalism (*b* = − 1.09, *SE* = 0.32, *95% CI* = [− 1.72, − 0.46], *P* = 0.0009), benevolence (*b* = − 0.71, *SE* = 0.27, *95% CI* = [− 1.25, − 0.18], *P* = 0.009), traditionalism (*b* = − 1.32, *SE* = 0.39, *95% CI* = [− 2.08, − 0.56], *P* = 0.0009), and conformity (*b* = − 0.20, *SE* = 0.37, *95% CI* = [− 1.99, − 0.47], *P* = 0.002). We observed overlapping results when considering TripPM total scores, which were also associated with placing less value on traditionalism (*b* = − 1.75, *SE* = 0.47, *95% CI* = [− 2.69, − 0.82], *P* = 0.0003) and conformity (*b* = − 1.30, *SE* = 0.48, *95% CI* = [− 2.25, − 0.35], *P* = 0.008) but more highly valuing stimulation (*b* = 1.82, *SE* = 0.54, *95% CI* = [0.76, 2.89], *P* = 0.001). We did not replicate findings supporting a relationship between psychopathy and endorsement of power and hedonism using either scale. Results thus suggest divergent patterns of values may be associated with altruism and psychopathy.

Linear models containing OUS scores as outcome variables found no relationship between LSRP or TriPM total scores and either impartial beneficence or instrumental harm. However, placing less value on impartial beneficence was associated with higher scores on the TriPM meanness subscale (*b* = − 1.03, *SE* = 0.27, *95% CI* = [− 1.57, − 0.50], *P* = 0.0002; Table S5), and the LSRP primary psychopathy subscale (*b* = − 0.96, SE = 0.32, *95% CI* = [− 1.60, − 0.31], *P* = 0.004); no similar relationships were seen for instrumental harm.

## Discussion

In the first exploration of patterns of moral cognition that characterize individuals who have engaged in real-world extraordinary altruism, we found that extraordinary altruists are distinguished from other people only with respect to a narrow set of moral concerns: they are more concerned with the moral foundation of harm/care, and they more strongly endorse impartial beneficence. Together, these findings support the conclusion that extraordinary altruists are morally motivated by an impartial concern for relieving suffering, and in turn, are motivated to improve others’ welfare in a self-sacrificial manner that does not allow for the harm of others in the process. These results are also partially consistent with extraordinary altruism representing the inverse of psychopathy in terms of moral cognition: altruists score lower in psychopathy (with the strongest relationships observed for psychopathy subscales associated with socio-affective responding) and higher-psychopathy participants most reliably endorse harm/care less than lower psychopathy participants, with participants with higher scores on the socio-affective subscales of our psychopathy measures also endorsing impartial beneficence less strongly.

These findings may yield important insights into the emergence of altruism more generally. Measuring altruism within the confines of the laboratory is notoriously difficult. Social desirability and norm-adherence motives can confound self-reported altruism, which does not reliably correspond to altruistic behavior^2^. And ethical and practical considerations prevent genuinely costly altruism that could incur risks or costs from being elicited by experimenters. Operationalizing altruism in terms of stringently defined real-world acts such as the donation of a kidney to an anonymous stranger helps to reduce these potential confounds. It should also be noted that this study was not aimed at prospectively *predicting* extraordinary altruism, an infeasible task given the extreme rarity of such donations, but rather identifying patterns of moral cognition that characterize real-world extraordinary altruists. It could be argued that observed group differences are caused by altruists’ prior donations. However, evidence of increased lifetime altruistic behaviors across a range of settings in altruistic kidney donors^3,4^ is more consistent with the conclusion that altruistic kidney donations reflect general traits that promote various forms of altruism—namely heightened care about others who are experiencing suffering or harm.

We predicted that extraordinary altruists would endorse individualizing foundations more strongly than controls, a prediction confirmed for the harm/care foundation. On average, altruists scored 0.23 points higher in harm/care compared to controls (see Supplementary Table S2 online), a substantial difference comparable in magnitude to the group difference between US liberals and conservatives (*M*_Liberals_ – *M*_Conservatives_ = 0.45;^50^), WEIRD and non-WEIRD samples (*M*_WEIRD_ – *M*_Non-WEIRD_ = 0.38;^11^), and Americans and Koreans (*M*_Americans_ – *M*_Koreans_ = 0.24;^51^). By contrast, altruists and controls did not differ in their valuation of fairness/reciprocity, although individualizing values of harm/care and fairness/reciprocity often track together^12–14^. This suggests that extraordinary altruism is not motivated by fairness/reciprocity concerns, such as alleviating the unfairness of altruists themselves having two functioning kidneys and their recipient having none. Rather, people who perform extraordinarily altruistic acts seem to be primarily motivated by alleviating suffering.

Consistent with this, and consistent with our predictions, altruists also endorsed impartial beneficence more than controls—although note that both altruists and controls scored higher (*M*_*Altruists*_ = 4.60, *M*_*Controls*_ = 3.98) than Kahane and colleagues’ MTurk sample (*M*_*Study1*_ = 3.75; *M*_*Study2*_ = 3.65;^36^). By contrast, we found no group differences in endorsement of instrumental harm. It is perhaps not surprising that endorsements of impartial beneficence distinguished altruists and controls, given that one of the questions on this scale is: “From a moral point of view, we should feel obliged to give one of our kidneys to a person with kidney failure since we don’t need two kidneys to survive, but really only one to be healthy”^37^. We confirmed that our findings were not driven by this item using analyses run without this item, which yielded similar and statistically significant results (see Supplementary Table S6 online). Taken together, these findings suggest that extraordinary altruists do not possess a strictly utilitarian approach to all moral dilemmas. Rather, they are motivated to act in a utilitarian manner if they can alleviate suffering without harming others in the process–consistent with our findings regarding their heightened concern for harm/care.

Because extraordinary altruists perform costly and risky acts to benefit strangers, we also predicted that they would express lower endorsement of binding foundations such as ingroup/loyalty, and purity/sanctity, but we found no group differences in these foundations. Thus, costly altruism towards strangers may not come at the cost of a reduced sense of concern for the ingroup. And although donating a kidney involves violating the wholeness of the body, the lack of group differences regarding purity/sanctity suggests that altruistic kidney donation also does not reflect a lack of concern for bodily violations. Taken together, our findings suggest that extraordinary altruists are distinguished from controls only with respect to harm/care relative to controls, but that they otherwise do not differ from controls in values that are related to ingroup concerns or political affiliation (suggesting that differences in political affiliation are unlikely to be driving observed group differences). That higher endorsements for harm/care does not come at the cost of the ingroup suggests that extraordinary altruists have an expanded circle of moral concern without this expansion coming at the cost of ingroup obligations.

Altruists’ increased concerns regarding harm and suffering are consistent with their lower psychopathy scores, particularly on subscales focusing on socio-affective components of psychopathy, such as the meanness scale of the TriPM meanness and the LSRP primary subscale. Meanwhile, less socially relevant psychopathic traits were not predictive of extraordinary altruism. This finding that the outward-facing, other-oriented dimension(s) of psychopathy is a significant negative predictor of extraordinary altruism, accompanied by the finding that altruists more strongly endorse considerations related to harm/care, paints a picture of altruistic individuals who are socially motivated to care for those around them. That altruists are no different than controls in terms of psychopathic traits associated with disinhibition and risk-taking offers some evidence that altruists did not choose to donate a kidney purely because they are bold, impulsive, or uninhibited. This interpretation is consistent with the findings we replicated relevant to psychopathy in our sample, such as that psychopathic traits predict lower endorsements of universalism and benevolence and lower concern for the moral foundation of harm/care.

Notably, and contrary to our predictions, we did not find that donating a kidney to a stranger is strongly or consistently correlated (positively or negatively) with basic values^27^ like universalism, benevolence, power, hedonism, or conformity. That suggests extraordinary altruism may not be driven by unusual values, at least as they are measured by the Schwartz inventory, but rather by specific moral concerns (such as harm/care). Our findings suggest that reported values may not in themselves predict whether one acts on those values when it comes to extraordinary altruism, much as “…a person can *value* being outgoing in social gatherings, independently of whether they are prone to acting in a lively or sociable manner”^52^. Similarly, people who share a common culture may value common things but acting on those values to an extraordinarily costly and altruistic degree may require a stronger motivation––a moral motivation.

The differences we observed between altruists and controls in terms of their moral values are sometimes reflected in altruists’ explanations of why they donated. When asked in interviews our lab has conducted about why they decided to donate, altruistic kidney donors have responded with answers like “…it was an instant no-brainer,” or described the decision by saying, “It felt comfortable and natural and fast”^53^. It is relatively rare for donors to report that any explicit or conscious moral reasoning or evaluation contributed to their judgment. More often they report that the decision was rapid and intuitive, in line with prevalent theories of prosocial responding^54^. This suggests that altruists’ pre-existing values related to the importance of helping those who are suffering prepare altruists to make relatively fast and automatic decisions about helping in relevant contexts when they learn about others who are suffering or in need of help.

Though constructs like harm/care and impartial beneficence seem on their face to overlap with the values of universalism and benevolence, past work has shown that Moral foundations and Schwartz values and are not one and the same^50^. Both universalism and benevolence are defined by Schwartz as having to do with welfare––universalism is defined as “understanding, appreciation, tolerance, and protection for the welfare of all people and for nature”^29^ and benevolence as “preserving and enhancing the welfare of those with whom one is in frequent personal contact”^29^. But a closer look at the items comprising these values show that concern for the welfare of others and concerns about harm reduction are only touched upon indirectly by the items. Universalism’s items ask about the importance of “equality”, “social justice”, “world at peace”, “wisdom”, “a world of beauty”, “protecting the environment”, “unity with nature”, and being “broadminded”^29^. Benevolence’s items ask about the importance of “true friendship”, being “loyal”, being “helpful, being “forgiving”, and being “honest”^29^. Meanwhile, the Harm/care foundation’s items more directly tap into concern for others’ welfare, with items asking for the moral relevance of “whether or not someone suffered emotionally” and whether: “Compassion for those who are suffering is the most crucial virtue.”^8^. Similarly, the OUS more directly assesses welfare valuation in its impartial beneficence subscale with items such as: “From a moral perspective, people should care about the well-being of all human beings on the planet equally; they should not favor the well-being of people who are especially close to them either physically or emotionally.” This important distinction between Harm/care and impartial benevolence and the Schwartz values of universalism/benevolence is corroborated by our correlational findings showing that the Schwartz values of universalism and benevolence are more strongly correlated with each other (*r* = 0.45; see Supplementary Table S3 online for all bivariate correlations) than they are correlated with any moral foundations or utilitarianism subscales.

Our null findings contradict prior findings from studies using self-report measures, which find everyday altruism to be positively associated with valuing universalism and negatively associated with valuating power^52^. This may reflect extraordinary altruism being motivated by distinct factors from those that motivate everyday prosociality^4^. Alternatively, it may reflect inherent limitations of measuring altruism and related variables using self-report scales, answers to which reflect social desirability and self-enhancement concerns in addition to altruistic motivation^2^. Our findings also raise the question: do extraordinary altruist see the world, morally speaking, in the same way as others, but are just more morally motivated to act on their beliefs, or do they see things differently and therefore act differently?

One unexpected finding was that extraordinary altruists reported valuing traditionalism (“respect, commitment, and acceptance of the customs and ideas that one’s culture or religion provides”^29^) more than controls did, but valued security (“safety, harmony, and stability of society, of relationships, and of self”^29^) less than controls. A potential explanation for these findings can be found when taking a closer look at the items that make up traditionalism and security. Though traditionalism’s definition is centered on customs, the items themselves ask about the importance of being “humble”, “moderate”, and accepting of one’s “portion in life”, among others^29^. This is consistent with recent work showing that extraordinary altruists are higher in humility compared to demographically similar control^55^. Similarly, though security’s definition is centered on safety, harmony, and stability, the items themselves ask about the importance of “social order”, being “clean” and “national security”, and among others^29^. At face value, some of these items composing security seem similar to the moral foundations’ binding values of Ingroup/loyalty, Authority/respect, and Purity/sanctity. And indeed, these binding values were all positively correlated with security (*r* > 0.50; see Supplementary Table S3 online for all bivariate correlations).

Our findings should be interpreted in light of some limitations. Our sample was largely white, consistent with the fact that 92% of altruistic kidney donors in the United States are white^1^. White adults may be overrepresented among the population of altruistic kidney donors in North America in part because they have increased access to health care^56^ and are less likely to possess medical risk factors that would disqualify them as kidney donors, such as high blood pressure or diabetes^57,58^. They also report higher trust in the health care system^59^, and are less concerned about discrimination in hospitals^60^. Notably, other populations of extraordinary altruists may be more diverse; for example, Black adults are overrepresented among heroic rescuers^61^. Our measures also do not exhaust the possible range of instruments measuring moral beliefs and values. We utilized the Oxford Utilitarianism Scale to test our preregistered hypotheses, but less consequentialist modes of moral judgments could be valuable to explore, such as deontological judgments^62^. Lastly, our mixed success in replicating associations between psychopathic traits and values^28^, moral foundations^44^, and utilitarian judgments^37^ should be interpreted in light of our sample’s low levels of psychopathy, as both altruistic kidney donors and controls who volunteer for psychology studies tend to be more prosocial than the general population^4,63^. Our findings thus should be interpreted as reflecting how psychopathic traits predict measures of values and moral judgments in populations with relatively low levels of psychopathy.

Despite these limitations, the present findings advance our understanding of the intersection of morality and altruism through the discovery that extraordinary altruists are not marked by a unique set of values or a strictly utilitarian approach to morality—that is, they are not simply real-life embodiments of utilitarianism, although they are often valorized by prominent utilitarians^64^ and the broadly utilitarian movement of ‘effective altruism’. Rather, altruists are marked primarily by heightened and relatively impartial concern for those suffering and in need. Our findings extend current literature on moral psychology to an ecologically-valid population of altruistic individuals, and add to current replication efforts, shedding light on which past findings regarding the relationship between psychopathic traits and values, moral foundations, and moral judgments hold true for a highly altruistic sample. Future studies should continue to investigate the moral psychology of altruistic kidney donors and other similarly altruistic groups, as increasing altruistic behavior will first require accurately understanding which moral mechanisms drive it.

## Methods

### Ethics and consent

This research protocol was approved by the Institutional Review Board of Georgetown University. The study was performed in accordance with approved guidelines and regulations, and informed consent was provided by all participants.

### Participants

We aimed to recruit 120 altruistic kidney donors and controls following an a priori power analysis conducted using G*Power version 3.1^65^ with parameters drawn from Glenn and colleagues’^28^ bivariate correlations between overall psychopathic traits scores and values from the Schwartz Values Scale^27^. To attain a more conservative estimate, we selected the smallest of the statistically significant effect sizes, which was the bivariate correlation between hedonism and overall psychopathic traits score. Using the parameters of α = 0.05, power = 0.80 and *r* = 0.28, the minimum sample required sample size was determined to be *n* = 99, which we increased to 120 (60 altruists, 60 controls) to allow for attrition or data collection errors. Participants were recruited through our database of verified altruistic kidney donors in North America and demographically similar controls. Participants who agreed to participate completed a survey using Qualtrics.

Of 120 recruited participants, one control failed to pass our attention check (planned exclusions included participants who failed ≥ 2 of 3 attention check items), and another participant originally recruited as a control who later became an altruistic kidney donor. Therefore, our final sample consisted of 61 altruists and 58 controls matched in age, gender, and race/ethnicity (see Supplementary Table S1 online). Participants were paid $10 electronically via PayPal.

### Materials

#### Moral foundation questionnaire (MFQ)

The MFQ^50^ gauges endorsement of concerns that people consider morally relevant. The 15-item scale contains five subscales: Harm/care, Fairness/reciprocity, Ingroup/loyalty, Authority/respect, and Purity/sanctity. Participants rate items using a 6-point scale from 0, “not at all relevant,” to 5, “extremely relevant”.

#### Schwartz values scale (SVS)

The SVS^27^ measures the importance of ten values (power, achievement, hedonism, stimulation, self-direction, universalism, benevolence, traditionalism, conformity, and security). Participants first are presented with 30 items and rate the importance of each value as a guiding principle on a scale of − 1 (opposed to my values) to 7 (of supreme importance). Then participants view 28 items phrased as ways of acting and asked participants to rate how important each item is as a guiding principle in their life on a scale of − 1 (opposed to my values) to 7 (of supreme importance).

#### Oxford utilitarianism scale (OUS)

The OUS indexes negative and positive dimensions of utilitarian judgments^37^ using 9 items scored on a 7-point scale. The OUS has two subscales, Impartial Beneficence (impartial maximization of the greater good, even at a personal cost) and Instrumental Harm (willingness to cause harm to bring about the greater good).

#### Triarchic psychopathy measure (TriPM)

The TriPM (Patrick, Fowles, & Krueger, 2009) measures three facets of psychopathy: boldness, meanness, and disinhibition; prior work has indicated altruists to be lower in meanness using a similar scale^4,45^. The scale contains 58 self-report items using a 4-point (false, somewhat false, somewhat true, true) scale. This measure is consistent and reliable with other measures of psychopathy^66–68^.

#### Levenson self-report psychopathy (LSRP)

Psychopathy was also measured using the LSRP^68^, a 26-item self-report measure composed of two factors: social and affective features such as manipulativeness, callousness, and lack of remorse, and antisocial features such as impulsiveness, irresponsibility, and sensation seeking. This measure has been well-validated in community samples[^69,70^], including those used to assess endorsement of basic values and moral foundations^28,44^.

#### Additional measures

Participants also completed the Social Dominance Orientation scale^71^ the Social Connectedness Scale—Revised^72^, and the UCLA Loneliness Scale—Version 3^73^. Though our preregistered analysis plan included exploratory analyses of these measures, results of these measures are not reported here because they (1) address questions that are outside the scope of this paper and (2) are not attached to any of our preregistered hypotheses. Participants indicated their attention using three attention checks^74^ distributed throughout the survey.

Finally, participants provided socio-demographic information (including age, gender, race, and education).

## Supplementary Information

Supplementary Information.

## Data Availability

De-identified data, analysis, preregistration, and study materials are available at: https://osf.io/xk4va/?view_only=4faad13131eb400983f065e3e2d9a22e.

## References

[1] US Dept. of Health. National Data. *Organ Procurement and Transplantation Network* https://optn.transplant.hrsa.gov/data/view-data-reports/national-data/# (2022).

[2] Brethel-Haurwitz KM, Stoycos SA, Cardinale EM, Huebner B, Marsh AA (2016). Is costly punishment altruistic? Exploring rejection of unfair offers in the ultimatum game in real-world altruists. Sci. Rep..

[3] Henderson AJ (2003). The living anonymous kidney donor: Lunatic or saint?. Am. J. Transplant..

[4] Vekaria KM, Brethel-Haurwitz KM, Cardinale EM, Stoycos SA, Marsh AA (2017). Social discounting and distance perceptions in costly altruism. Nat. Hum. Behav..

[5] Skitka LJ (2010). The psychology of moral conviction. Soc. Personal. Psychol. Compass.

[6] Ellemers N, Van Der Toorn J, Paunov Y, Van Leeuwen T (2019). The psychology of morality: A review and analysis of empirical studies published from 1940 through 2017. Pers. Soc. Psychol. Rev..

[7] Fiske AP, Tetlock PE (1997). Taboo trade-offs: Reactions to transactions that transgress the spheres of justice. Polit. Psychol..

[8] Haidt J, Graham J (2007). When morality opposes justice: Conservatives have moral intuitions that liberals may not recognize. Soc. Justice Res..

[9] Koleva SP, Graham J, Iyer R, Ditto PH, Haidt J (2012). Tracing the threads: How five moral concerns (especially Purity) help explain culture war attitudes. J. Res. Pers..

[10] Alberici AI, Milesi P (2016). Online discussion, politicized identity, and collective action. Group Process. Intergroup Relat..

[11] Doğruyol B, Alper S, Yilmaz O (2019). The five-factor model of the moral foundations theory is stable across WEIRD and non-WEIRD cultures. Pers. Individ. Differ..

[12] Graham J, Haidt J, Nosek BA (2009). Liberals and conservatives rely on different sets of moral foundations. J. Pers. Soc. Psychol..

[13] Napier JL, Luguri JB (2013). Moral mind-sets: Abstract thinking increases a preference for “individualizing” over “binding” moral foundations. Soc. Psychol. Personal. Sci..

[14] Clark CB (2017). A behavioral economic assessment of individualizing versus binding moral foundations. Pers. Individ. Differ..

[15] Joseph CM, Graham J, Haidt J (2009). The end of equipotentiality: A moral foundations approach to ideology-attitude links and cognitive complexity. Psychol. Inq..

[16] Waytz A, Dungan J, Young L (2013). The whistleblower's dilemma and the fairness–loyalty tradeoff. J. Exp. Soc. Psychol..

[17] Graham J, Waytz A, Meindl P, Iyer R, Young L (2017). Centripetal and centrifugal forces in the moral circle: Competing constraints on moral learning. Cogn..

[18] Singer N, Kreuzpointner L, Sommer M, Wüst S, Kudielka BM (2019). Decision-making in everyday moral conflict situations: Development and validation of a new measure. PLoS ONE.

[19] Tetlock PE, Armor D, Peterson RS (1994). The slavery debate in antebellum America: Cognitive style, value conflict, and the limits of compromise. J. Pers. Soc. Psychol..

[20] Everett JA, Faber NS, Savulescu J, Crockett MJ (2018). The costs of being consequentialist: Social inference from instrumental harm and impartial beneficence. J. Exp. Soc. Psychol..

[21] Stothers L, Gourlay WA, Liu L (2005). Attitudes and predictive factors for live kidney donation: A comparison of live kidney donors versus nondonors. Kidney Int..

[22] Rios A (2010). Evaluation of attitudes toward living organ donation: A multicenter study of compulsory secondary school education teachers. Transplant. Proc..

[23] Tong A (2012). The motivations and experiences of living kidney donors: A thematic synthesis. Am. J. Kidney Dis..

[24] Süssenbach P, Rees J, Gollwitzer M (2019). When the going gets tough, individualizers get going: On the relationship between moral foundations and prosociality. Pers. Individ. Differ..

[25] Nilsson A, Erlandsson A, Västfjäll D (2020). Moral foundations theory and the psychology of charitable giving. Eur. J. Pers..

[26] Shepherd AM, Schnitker SA, Greenway TS (2019). Religious service attendance, moral foundations, god concept, and in-group giving: Testing moderated mediation. Rev. Relig. Res..

[27] Schwartz SH (1992). Universals in the content and structure of values: Theoretical advances and empirical tests in 20 countries. Adv. Exp. Soc. Psychol..

[28] Glenn AL, Efferson LM, Iyer R, Graham J (2017). Values, goals, and motivations associated with psychopathy. J. Soc. Clin. Psychol..

[29] Schwartz SH (2012). An overview of the Schwartz theory of basic values. Online Read. Psychol. Culture.

[30] Feldman G (2021). Personal values and moral foundations: Examining relations and joint prediction of moral variables. Soc. Psychol. Personal. Sci..

[31] Lönnqvist JE, Verkasalo M, Wichardt PC, Walkowitz G (2013). Personal values and prosocial behaviour in strategic interactions: Distinguishing value-expressive from value-ambivalent behaviours. Eur. J. Soc. Psychol..

[32] Bardi A, Schwartz SH (2003). Values and behavior: Strength and structure of relations. Pers. Soc. Psychol. Bull..

[33] Persson BN, Kajonius PJ (2016). Empathy and universal values explicated by the empathy-altruism hypothesis. J. Soc. Psychol..

[34] Greene JD, Sommerville RB, Nystrom LE, Darley JM, Cohen JD (2001). An fMRI investigation of emotional engagement in moral judgment. Sci..

[35] Greene JD (2007). Why are VMPFC patients more utilitarian? A dual-process theory of moral judgment explains. Trends Cogn. Sci..

[36] Greene JD, Nystrom LE, Engell AD, Darley JM, Cohen JD (2004). The neural bases of cognitive conflict and control in moral judgment. Neuron.

[37] Kahane G (2018). Beyond sacrificial harm: A two-dimensional model of utilitarian psychology. Psychol. Rev..

[38] Bentham J (1789). A utilitarian view. Animal Rights Hum. Oblig..

[39] Mill JS (1863). Utilitarianism.

[40] Singer P (2011). Practical Ethics.

[41] Fowler Z, Law KF, Gaesser B (2021). Against empathy bias: The moral value of equitable empathy. Psychol. Sci..

[42] Blair RJR (2001). Neurocognitive models of aggression, the antisocial personality disorders, and psychopathy. J. Neurol. Neurosurg. Psychiatry.

[43] Hare RD, Neumann CS (2005). Structural models of psychopathy. Curr. Psychiatry Rep..

[44] Glenn AL, Iyer R, Graham J, Koleva S, Haidt J (2009). Are all types of morality compromised in psychopathy?. J. Pers. Disord..

[45] Marsh AA (2014). Neural and cognitive characteristics of extraordinary altruists. Proc. Natl. Acad. Sci..

[46] O’Connell K (2019). Increased similarity of neural responses to experienced and empathic distress in costly altruism. Sci. Rep..

[47] Berluti K (2020). Reduced multi-voxel pattern similarity of vicarious neural pain responses in psychopathy. J. Pers. Disord..

[48] McFadden D (1974). The measurement of urban travel demand. J. Public Econ..

[49] Schwartz SH (2009). Draft users’ Manual: Proper use of the Schwarz value survey. Cross Cult. Strateg. Manag..

[50] Graham J (2011). Mapping the moral domain. J. Pers. Soc. Psychol..

[51] Kim KR, Kang J-S, Yun S (2012). Moral intuitions and political orientation: Similarities and differences between South Korea and the United States. Psychol. Rep..

[52] Anglim J, Knowles ER, Dunlop PD, Marty A (2017). HEXACO personality and Schwartz's personal values: A facet-level analysis. J. Res. Pers..

[53] Marsh, A. A. (2015). *Altruistic kidney donor interviews.* Internal Laboratory on Social and Affective Neuroscience report: unpublished.

[54] Rand DG, Epstein ZG (2014). Risking your life without a second thought: Intuitive decision-making and extreme altruism. PLoS ONE.

[55] Rhoads, S. A., Vekaria, K., OConnell, K., Elizabeth, H. S., Rand, D., Williams, M. K., & Marsh, A. Unselfish traits and social decision-making patterns characterize six populations of real-world extraordinary altruists. 10.31234/osf.io/sykmv (Under review). 10.1038/s41467-023-37283-5 PMC10066349 37002205

[56] Carratala, S. & Maxwell, C. Health disparities by race and ethnicity. *American Progress* https://www.americanprogress.org/article/health-disparities-race-ethnicity/ (2020).

[57] Lackland DT (2014). Racial differences in hypertension: Implications for high blood pressuremanagement. Am. J. Med. Sci..

[58] Harris MI (1998). Prevalence of diabetes, impaired fasting glucose, and impaired glucose tolerance in US adults: The Third National Health and Nutrition Examination Survey, 1988–1994. Diabetes Care.

[59] Siminoff LA, Burant CJ, Ibrahim SA (2006). Racial disparities in preferences and perceptions regarding organ donation. J. Gen. Intern. Med..

[60] Boulware LE (2002). Understanding disparities in donor behavior: Race and gender differences in willingness to donate blood and cadaveric organs. Med. Care.

[61] Zimbardo, P. What makes a hero? *Greater Good Magazine* https://greatergood.berkeley.edu/article/item/what_makes_a_hero (2011).

[62] Levine S, Kleiman-Weiner M, Schulz L, Tenenbaum J, Cushman F (2020). The logic of universalization guides moral judgment. Proc. Natl. Acad. Sci..

[63] Van Lange PA, Schippers M, Balliet D (2011). Who volunteers in psychology experiments? An empirical review of prosocial motivation in volunteering. Pers. Individ. Differ..

[64] Singer, P. *The why and how of effective altruism* [Video]. TED Conferences. https://www.ted.com/talks/peter_singer_the_why_and_how_of_effective_altruism?language=en (2013).

[65] Faul F, Erdfelder E, Buchner A, Lang A-G (2009). Statistical power analyses using G*Power 3.1: Tests for correlation and regression analyses. Behav. Res. Methods.

[66] Drislane LE, Patrick CJ, Arsal G (2014). Clarifying the content coverage of differing psychopathy inventories through reference to the Triarchic Psychopathy Measure. Psychol. Assess..

[67] Patrick CJ, Drislane LE (2014). Triarchic model of psychopathy: Origins, operationalizations, and observed linkages with personality and general psychopathology. J. Pers..

[68] van Dongen JDM, Drislane LE, Nijman H, Soe-Agnie SE, van Marle HJC (2017). Further evidence for the reliability and validity of the Triarchic Psychopathy Measure in a forensic and a community sample. J. Psychopathol. Behav. Assess..

[69] Levenson MR, Kiehl KA, Fitzpatrick CM (1995). Assessing psychopathic attributes in a noninstitutionalized population. J. Pers. Soc. Psychol..

[70] Lynam DR, Whiteside S, Jones S (1999). Self-reported psychopathy: A validation study. J. Pers. Assess..

[71] Pratto F, Sidanius J, Stallworth LM, Malle BF (1994). Social dominance orientation: A personality variable predicting social and political attitudes. J. Pers. Soc. Psychol..

[72] Lee RM, Draper M, Lee S (2001). Social connectedness, dysfunctional interpersonal behaviors, and psychological distress: Testing a mediator model. J. Couns. Psychol..

[73] Russell DW (1996). UCLA Loneliness Scale (Version 3): Reliability, validity, and factor structure. J. Pers. Assess..

[74] Maniaci MR, Rogge RD (2014). Caring about carelessness: Participant inattention and its effects on research. J. Res. Pers..

