# Supplementary material for: Moral foundations, values, and judgments in extraordinary altruists
Source: Sci Rep. 2022 Dec 21;12:22111. doi: 10.1038/s41598-022-26418-1 (PMC9772189; doi:10.1038/s41598-022-26418-1)
Supplement: Supplementary file 1 — Supplementary Information. [file 41598_2022_26418_MOESM1_ESM.docx]

Moral Foundations, Values, and Reasoning in Extraordinary Altruists

Supplementary Materials

**Supplementary Table S1**

*Participant Demographics*

|  | Altruists | Controls | p-value |
| --- | --- | --- | --- |
| N | 61 | 58 |  |
| Age | 43.2 (11.4) | 42.9 (8.7) | 0.89 |
| Gender (% female) | 54.1% | 53.4% | 1.00 |
| Education (% ≥ 4-year degree) | 90.16% | 96.55% | 0.31 |
| Household Income (% ≥ $60,000) | 81.97% | 84.48% | 0.50 |
| Race / Ethnicity |  |  | 0.38 |
| White | 91.80% | 93.10% |  |
| Black / African American | 0% | 0% |  |
| Asian  Latino / Hispanic | 3.28%  3.28% | 5.17%  0% |  |
| Multiple / Other | 1.64% | 1.72% |  |

*Note*. Significance was obtained using 2-sided independent-samples t-tests for continuous variables and Pearson chi-square analyses for categorical variables.

**Supplementary Table S2**

*Descriptive Statistics*

|  | **Altruists** | | | **Controls** | | |
| --- | --- | --- | --- | --- | --- | --- |
|  | M (SD) | Min - Max | Skew (Kurtosis) | M (SD) | Min - Max | Skew (Kurtosis) |
| MFQ Harm | 3.89 (0.70) | 3.33-5.00 | -0.60 (3.08) | 3.66 (0.66) | 2.00-4.83 | -0.38 (2.88) |
| MFQ Fairness | 3.25 (0.68) | 1.50-4.50 | -0.63 (3.11) | 3.33 (0.68) | 1.83-4.33 | -0.62 (2.93) |
| MFQ Loyalty | 1.87 (0.91) | 0.17-4.17 | 0.09 (2.16) | 2.12 (0.77) | 0.67-3.83 | 0.20 (2.61) |
| MFQ Authority | 2.12 (1.00) | 0.00-4.50 | 0.03 (2.41) | 2.42 (0.94) | 0.17-4.50 | 0.01 (2.69) |
| MFQ Purity | 1.66 (1.09) | 0.00-4.50 | 0.47 (2.65) | 1.90 (1.23) | 0.00-4.67 | 0.40 (2.21) |
| OUS Impartial Beneficence | 4.60 (1.11) | 2.20-6.80 | -0.30 (2.59) | 3.98 (1.19) | 1.00-6.00 | -0.45 (2.77) |
| OUS Instrumental Harm | 3.20 (1.29) | 1.00-7.00 | 0.31 (3.01) | 3.16 (0.97) | 1.00-5.25 | -0.17 (2.41) |
| Power | 1.67 (1.34) | -0.80-5.60 | 0.58 (3.19) | 1.90 (1.19) | -0.20-4.40 | 0.25 (2.45) |
| Achievement | 4.11 (1.19) | 0.75-7.00 | -0.01 (2.90) | 4.19 (1.20) | 1.50-6.25 | -0.38 (2.35) |
| Hedonism | 4.13 (1.02) | 2.33-6.67 | 0.28 (2.54) | 3.93 (1.17) | 1.33-6.33 | 0.04 (2.31) |
| Stimulation | 3.50 (1.41) | 0.33-6.67 | -0.04 (2.64) | 3.44 (1.62) | -0.33-6.67 | 0.00 (2.16) |
| Self-direction | 5.24 (1.07) | 2.60-7.40 | -0.02 (2.34) | 5.27 (1.00) | 3.00-7.20 | -0.36 (2.58) |
| Universalism | 4.51 (0.97) | 1.38-6.38 | -0.39 (3.35) | 4.45 (1.20) | 1.12-6.38 | -0.70 (2.84) |
| Benevolence | 5.04 (0.88) | 3.00-6.60 | -0.42 (2.33) | 4.99 (0.96) | 2.40-6.60 | -0.36 (2.86) |
| Traditionalism | 4.22 (1.36) | 1.80-6.80 | 1.36 (0.17) | 3.99 (1.24) | 0.80-6.20 | -0.29 (2.56) |
| Conformity | 3.74 (1.46) | 0.25-6.25 | -0.31 (2.48) | 3.89 (1.20) | 1.00-6.75 | -0.02 (3.03) |
| Security | 3.64 (1.27) | -0.20-6.60 | -0.39 (3.65) | 4.13 (1.15) | 2.00-6.80 | 0.01 (2.49) |
| TriPM Boldness | 1.75 (0.45) | 0.47-2.68 | -0.37 (3.14) | 1.66 (0.50) | 0.32-2.68 | -0.20 (3.26) |
| TriPM Disinhibition | 0.52 (0.33) | 0.00-1.50 | 0.72 (3.41) | 0.63 (0.43) | 0.10-2.45 | 1.82 (7.45) |
| TriPM Meanness | 0.33 (0.27) | 0.00-1.11 | 1.04 (3.32) | 0.54 (0.47) | 0.00-2.16 | 1.19 (4.05) |
| TriPM Total | 0.86 (0.22) | 0.31-1.59 | 0.49 (3.93) | 0.94 (0.28) | 0.40-1.69 | 0.72 (3.30) |
| LSRP Primary | 0.45 (0.25) | 0.12-1.38 | 1.41 (5.24) | 0.64 (0.39) | 0.19-1.81 | 1.14 (3.61) |
| LSRP Secondary | 0.68 (0.45) | 0.00-1.80 | 0.97 (3.29) | 0.77 (0.42) | 0.10-2.10 | 0.90 (3.51) |
| LSRP Total | 0.54 (0.27) | 0.15-1.42 | 1.13 (4.17) | 0.69 (0.34) | 0.23-1.73 | 1.13 (4.17) |

**Supplementary Table S3**

*Bivariate Correlations using Spearman Correlation*

| Variable | 1 | 2 | 3 | 4 | 5 | 6 | 7 | 8 | 9 | 10 | 11 | 12 | 13 | 14 | 15 | 16 | 17 | 18 | 19 | 20 | 21 | 22 | 23 | 24 | 25 | 26 | 27 | 28 | 29 |
| --- | --- | --- | --- | --- | --- | --- | --- | --- | --- | --- | --- | --- | --- | --- | --- | --- | --- | --- | --- | --- | --- | --- | --- | --- | --- | --- | --- | --- | --- |
| 1. Altruism |  |  |  |  |  |  |  |  |  |  |  |  |  |  |  |  |  |  |  |  |  |  |  |  |  |  |  |  |  |
| 2. Power | -0.10 |  |  |  |  |  |  |  |  |  |  |  |  |  |  |  |  |  |  |  |  |  |  |  |  |  |  |  |  |
| 3. Achieve. | -0.06 | 0.56*** |  |  |  |  |  |  |  |  |  |  |  |  |  |  |  |  |  |  |  |  |  |  |  |  |  |  |  |
| 4. Hedonism | 0.08 | 0.18 | 0.23* |  |  |  |  |  |  |  |  |  |  |  |  |  |  |  |  |  |  |  |  |  |  |  |  |  |  |
| 5. Stimulation | 0.02 | 0.12 | 0.28** | 0.37*** |  |  |  |  |  |  |  |  |  |  |  |  |  |  |  |  |  |  |  |  |  |  |  |  |  |
| 6. Self-Direct. | -0.03 | 0.16 | 0.38*** | 0.37*** | 0.61*** |  |  |  |  |  |  |  |  |  |  |  |  |  |  |  |  |  |  |  |  |  |  |  |  |
| 7. Universal. | -0.01 | 0.1 | 0.31*** | 0.33*** | 0.42*** | 0.47*** |  |  |  |  |  |  |  |  |  |  |  |  |  |  |  |  |  |  |  |  |  |  |  |
| 8. Benevol. | 0.03 | 0.2* | 0.44*** | 0.54*** | 0.25** | 4.20E-01 | 0.45*** |  |  |  |  |  |  |  |  |  |  |  |  |  |  |  |  |  |  |  |  |  |  |
| 9. Traditional. | 0.06 | 0.28** | 0.23* | 0.06 | 0.01 | 0.11 | 0.33*** | 0.39*** |  |  |  |  |  |  |  |  |  |  |  |  |  |  |  |  |  |  |  |  |  |
| 10. Conform. | -0.03 | 0.4*** | 0.46*** | 0.14 | 0.06 | 0.25** | 0.29** | 0.52*** | 0.61*** |  |  |  |  |  |  |  |  |  |  |  |  |  |  |  |  |  |  |  |  |
| 11. Security | -0.2* | 0.56*** | 0.45*** | 0.23* | 0.14 | 0.33*** | 0.29** | 0.47*** | 0.41*** | 0.68*** |  |  |  |  |  |  |  |  |  |  |  |  |  |  |  |  |  |  |  |
| 12. OUS IB | 0.24** | -0.25** | -0.11 | 0.06 | 0.15 | -0.01 | 0.3** | 0.04 | 0.06 | -0.14 | -0.23* |  |  |  |  |  |  |  |  |  |  |  |  |  |  |  |  |  |  |
| 13. OUS IH | 0.01 | 0.15 | 0.04 | 0.10 | 0.12 | 0.01 | 0.03 | -0.08 | -0.01 | -0.15 | 0 | 0.15 |  |  |  |  |  |  |  |  |  |  |  |  |  |  |  |  |  |
| 14. Harm | 0.18 | -0.22* | 0.09 | 0.16 | 0.02 | 0.03 | 0.33*** | 0.19* | 0.13 | 0.14 | 0.04 | 0.35*** | -0.06 |  |  |  |  |  |  |  |  |  |  |  |  |  |  |  |  |
| 15. Fairness | -0.04 | 0.09 | 0.19* | 0.18 | 0.2* | 0.03 | 0.36*** | 0.27** | 0.12 | 0.17 | 0.18 | 0.29** | -0.03 | 0.45*** |  |  |  |  |  |  |  |  |  |  |  |  |  |  |  |
| 16. Loyalty | -0.13 | 0.5*** | 0.32*** | 0.01 | 0.05 | 0.04 | 0.05 | 0.17 | 0.21* | 0.53*** | 0.48*** | -0.13 | 0.03 | 0.02 | 0.24* |  |  |  |  |  |  |  |  |  |  |  |  |  |  |
| 17. Authority | -0.14 | 0.53*** | 0.32*** | -0.04 | -0.10 | 0.02 | 0.01 | 0.24** | 0.37*** | 0.63*** | 0.62*** | -0.33*** | -0.01 | -0.04 | 0.10 | 0.73*** |  |  |  |  |  |  |  |  |  |  |  |  |  |
| 18. Purity | -0.09 | 0.43*** | 0.25** | -0.07 | -0.21 | -0.07 | 0 | 0.18 | 0.46*** | 0.64*** | 0.5*** | -0.17 | -0.07 | 0.06 | 0.11 | 0.58*** | 0.73*** |  |  |  |  |  |  |  |  |  |  |  |  |
| 19. LSRP Tot. | -0.24* | 0.15 | -0.10 | 0 | 0.05 | -0.04 | -0.23* | -0.21* | -0.23* | -0.23* | -0.01 | -0.14 | -0.05 | -0.32*** | -0.21* | 0.10 | -0.03 | -0.04 |  |  |  |  |  |  |  |  |  |  |  |
| 20. LSRP 1 | -0.25** | 0.19* | 0 | 0.03 | 0.15 | 0.03 | -0.18 | -0.17 | -0.18 | -0.17 | 0 | -0.18 | 0.06 | -0.38*** | -0.23* | 0.13 | 0.01 | -0.11 | 0.83*** |  |  |  |  |  |  |  |  |  |  |
| 21. LSRP 2 | -0.14 | 0.10 | -0.09 | -0.07 | -0.01 | -0.05 | -0.14 | -0.17 | -0.13 | -0.17 | 0.03 | 0.01 | -0.1 | -0.19* | -0.09 | 0.09 | -0.04 | 0.07 | 0.78*** | 0.34*** |  |  |  |  |  |  |  |  |  |
| 22. Tri. Total | -0.13 | 0.18* | 0.18* | 0.03 | 0.34*** | 0.18* | -0.09 | -0.08 | -0.24** | -0.17 | 0.06 | -0.04 | 0.01 | -0.24** | -0.02 | 0.11 | -0.01 | -0.11 | 0.54*** | 0.54*** | 0.40*** |  |  |  |  |  |  |  |  |
| 23. Tri. Mean | -0.23* | 0.16 | -0.01 | -0.03 | 0.18* | 0.07 | -0.15 | -0.15 | -0.26** | -0.22* | 0.06 | -0.15 | 0.01 | -0.4*** | -0.15 | 0.10 | 0.02 | -0.08 | 0.68*** | 0.63*** | 0.49*** | 0.77*** |  |  |  |  |  |  |  |
| 24. Tri. Disinhi. | -0.11 | 0.11 | 0.02 | 0.05 | 0.02 | 0.02 | -0.11 | -0.09 | -0.14 | -0.11 | 0.08 | -0.11 | -0.07 | -0.09 | -0.06 | 0.15 | 0.08 | 0.01 | 0.66*** | 0.50*** | 0.62*** | 0.55*** | 0.50*** |  |  |  |  |  |  |
| 25. Tri. Bold. | 0.11 | 0.11 | 0.29** | 0.08 | 0.49*** | 0.24** | 0.17 | 0.09 | -0.09 | -0.01 | -0.05 | 0.21* | 0.10 | 0.05 | 0.16 | 0.01 | -0.12 | -0.11 | -0.16 | -0.01 | -0.20* | 0.49*** | 0.05 | 0.24** |  |  |  |  |  |
| 26. Age | 0 | -0.01 | 0 | -0.04 | 0.02 | 0.12 | 0.16 | 0.14 | 0.14 | 0.16 | 0.15 | 0.07 | -0.16 | 0.09 | 0.04 | 0.07 | 0.14 | 0.08 | -0.28** | -0.26** | -0.21* | -0.01 | -0.14 | -0.10 | 0.14 |  |  |  |  |
| 27. Education | 0.05 | -0.16 | -0.15 | -0.27** | -0.08 | -0.11 | 0 | -0.18 | -0.08 | -0.25** | -0.28** | 0.17 | 0.10 | -0.04 | -0.01 | -0.12 | -0.28** | -0.18* | -0.18 | -0.13 | -0.13 | -0.16 | -0.15 | -0.25** | 0.11 | 0.13 |  |  |  |
| 28. Gender ^a^ | -0.01 | 0.13 | 0.06 | 0.12 | 0.27** | 0.15 | 0.10 | -0.06 | 0.02 | -0.02 | 0.07 | 0.15 | 0.07 | -0.05 | 0.13 | 0.07 | -0.10 | -0.16 | 0.17 | 0.15 | 0.14 | 0.32*** | 0.25** | 0.04 | 0.25** | -0.08 | 0 |  |  |
| 29. Income | -0.11 | 0.14 | 0.10 | -0.13 | -0.03 | 0.01 | -0.09 | -0.02 | -0.12 | -0.06 | 0.10 | 0 | 0.08 | -0.11 | 0 | 0.10 | 0.06 | 0 | -0.11 | -0.06 | -0.11 | 0.11 | 0 | -0.07 | 0.19* | 0.29** | 0.11 | 0.14 |  |

*Note*. OUS IB/IH = Oxford Utilitarianism Scale, Impartial Beneficence/Instrumental Harm. Tri. = Triarchic Psychopathy Measure.

^a^ 0 = female; 1 = male

*^+^ p* < .1; * *p* < .05; ** *p* < .01; *** *p* < .001.

**Supplementary Table S4**

*Single Logistic Regression Predicting Altruist Status from Remaining Schwartz Values Controlling for Sociodemographic Variables*

| Factor | *b* (SE) | OR [CI 95%] |
| --- | --- | --- |
| Achievement | 0.07 (0.19) | 1.07 [0.73, 1.57] |
| Stimulation | 0.04 (0.17) | 1.04 [0.75, 1.46] |
| Self-direction | 0.06 (0.25) | 1.06 [0.65, 1.74] |
| Traditionalism | 0.36 (0.17)* | 1.43 [1.02, 2.01] |
| Security  *Demographic covariates* | -0.61 (0.22)** | 0.54 [0.35, 0.83] |
| Age | 0.01 (0.02) | 1.01 [0.97, 1.05] |
| Education | -0.11 (0.21) | 0.90 [0.59, 1.36] |
| Gender *^a^* | 0.01 (0.40) | 1.01 [0.46, 2.22] |

*Note.* Altruists were coded as ‘1’ and controls were coded as ‘0’.

*^a^* 0 = female; 1 = male.

*^+^ p* < .1; * *p* < .05; ** *p* < .01; *** *p* < .001.

**Supplementary Table S5**

*Multiple Linear Regressions of Psychopathic Trait Scores Predicting Study Variables, Controlling for Sociodemographic Variables*

| Factor | Triarchic | Psychopathy | Measure (TriPM) | | Levenson Self-Report Psychopathy | | (LSRP) |
| --- | --- | --- | --- | --- | --- | --- | --- |
|  | **TriPM Boldness**  ***b* (SE)** | **TriPM Disinhibition**  ***b* (SE)** | **TriPM Meanness**  ***b* (SE)** | **TriPM Total**  ***b* (SE)** | **LSRP Primary**  ***b* (SE)** | **LSRP Secondary**  ***b* (SE)** | **LSRP Total**  ***b* (SE)** |
| Harm | 0.05 (0.14) | -0.32 (0.17) *^+^* | -0.82 (0.15)*** | -0.87 (0.25)** | -0.84 (0.18)*** | -0.36 (0.15)* | -0.89 (0.20)*** |
| Fairness | 0.16 (0.13) | -0.15 (0.16) | -0.40 (0.15)** | -0.76 (0.24) | -0.54 (0.17)** | -0.17 (0.14) | -0.53 (0.19)** |
| Loyalty | -0.03 (0.17) | 0.23 (0.21) | 0.18 (0.21) | 0.30 (0.33) | 0.19 (0.24) | 0.03 (0.19) | 0.16 (0.26) |
| Authority | -0.25 (0.19) | 0.03 (0.23) | 0.21 (0.23) | -0.09 (0.36) | -0.05 (0.27) | -0.21 (0.20) | -0.21 (0.29) |
| Purity | -0.27 (0.23) | 0.06 (0.28) | -0.09 (0.28) | -0.33 (0.44) | -0.31 (0.32) | 0.04 (0.25) | -0.20 (0.35) |
| Power | 0.19 (0.26) | 0.18 (0.31) | 0.19 (0.31) | 0.52 (0.48) | 0.43 (0.36) | -0.06 (0.27) | 0.27 (0.39) |
| Achievement | 0.83 (0.23)*** | -0.15 (0.30) | -0.27 (0.29) | 0.62 (0.46) | -0.48 (0.34) | -0.49 (0.26) *^+^* | -0.73 (0.36)* |
| Hedonism | 0.22 (0.22) | -0.02 (0.26) | -0.42 (0.25) *^+^* | -0.12 (0.41) | 0.12 (0.30) | -0.13 (0.23) | -0.01 (0.33) |
| Stimulation | 1.37 (0.27)*** | 0.03 (0.36) | 0.26 (0.36) | 1.82 (0.54)*** | 0.33 (0.42) | -0.07 (0.32) | 0.18 (0.46) |
| Self-direction | 0.44 (0.21)* | -0.01 (0.25) | -0.04 (0.25) | 0.47 (0.39) | -0.17 (0.29) | -0.15 (0.22) | -0.24 (0.32) |
| Universalism | 0.26 (0.22) | -0.35 (0.27) | -0.89 (0.25)*** | -0.70 (0.41) *^+^* | -1.06 (0.29)*** | -0.40 (0.23) *^+^* | -1.09 (0.32)*** |
| Benevolence | 0.22 (0.18) | -0.33 (0.22) | -0.54 (0.21)* | -0.46 (0.34) | -0.59 (0.25)* | -0.36 (0.19) *^+^* | -0.71 (0.27)** |
| Traditionalism | -0.32 (0.26) | -0.74 (0.32)* | -0.95 (0.31)** | -1.75 (0.47)*** | -1.16 (0.35)** | -0.59 (0.28)* | -1.32 (0.39)*** |
| Conformity | -0.14 (0.26) | -0.59 (0.31) *^+^* | -0.80 (0.30)* | -1.30 (0.48)** | -0.87 (0.36)* | -0.75 (0.27)** | -0.20 (0.37)** |
| Security | -0.28 (0.24) | -0.05 (0.29) | -0.80 (0.30) | -0.33 (0.45) | -0.13 (0.34) | -0.14 (0.26) | -0.20 (0.37) |
| Impartial Beneficence | 0.44 (0.24) *^+^* | -0.31 (0.29) | -1.03 (0.27)*** | -0.59 (0.45) | -0.96 (0.32)** | -0.05 (0.26) | -0.74 (0.36) |
| Instrumental Harm | 0.39 (0.23) *^+^* | -0.18 (0.28) | -0.28 (0.28) | 0.07 (0.44) | 0.03 (0.32) | -0.28 (0.24) | -0.20 (0.35) |

*Note.* Beta values and standard errors are from multiple regression models predicting Moral Foundations, Schwartz values, and Oxford Utilitarianism subscales, from psychopathy scores while controlling for age, sex, and education.

*^+^ p* < .1; * *p* < .05; ** *p* < .01; *** *p* < .001.

**Supplementary Table S6**

*Results from Single Logistic Regression Predicting Altruist Status from Oxford Utilitarianism Subscales with Kidney Donation Item Removed from Impartial Beneficence Subscale*

| Factor | *b* (SE) | OR [CI 95%] |
| --- | --- | --- |
| Impartial Beneficence *^a^* | 0.42 (0.16)** | 1.53 [1.11, 2.10] |
| Instrumental Harm  *Demographic Covariates* | -0.02 (0.17) | 0.98 [0.70, 1.37] |
| Age | -0.001 (0.02) | 1.00 [0.96, 1.04] |
| Education | -0.05 (0.20) | 0.95 [0.64, 1.42] |
| Gender *^b^* | -0.14 (0.38) | 0.87 [0.41, 1.84] |

*Note.* Altruists were coded as ‘1’ and controls were coded as ‘0’.

*^a^* Subscale recalculated as the average of the four remaining items from Impartial Beneficence, without the fifth item regarding kidney donation.

*^b^* 0 = female; 1 = male.

*^+^ p* < .1; * *p* < .05; ** *p* < .01; *** *p* < .001.
